# Application of Organic Nanofibers to Boost Specialized Metabolite Production and Antioxidant Potential in *Stevia rebaudiana* In Vitro Cultures

**DOI:** 10.3390/metabo15090579

**Published:** 2025-08-29

**Authors:** Maria Geneva, Antoaneta Trendafilova, Kamelia Miladinova-Georgieva, Mariana Sichanova, Daniela Tsekova, Viktoria Ivanova, Elisaveta Kirova, Maria Petrova

**Affiliations:** 1 Institute of Plant Physiology and Genetics, Bulgarian Academy of Sciences, 1113 Sofia, Bulgaria; mariqg@gmail.com (M.G.);; 2 Institute of Organic Chemistry with Centre of Phytochemistry, Bulgarian Academy of Sciences, 1113 Sofia, Bulgaria; antoaneta.trendafilova@orgchm.bas.bg (A.T.);; 3 Department of Organic Chemistry, University of Chemical Technology and Metallurgy, 1756 Sofia, Bulgaria

**Keywords:** *Stevia rebaudiana* Bert., in vitro propagation, antioxidant activity, organic nanofibers, steviol glycosides, caffeoylquinic acids

## Abstract

**Background:** Potential advantages for improving plant growth, stress tolerance, and valuable metabolites generation are provided by the implementation of nanotechnology into plant biotechnology. A recently discovered technique with significant promise for agricultural practices is the use of biopolymer-based nanomaterials, like peptidomimetics, as insecticides, growth regulators, and nutrient carriers. This study explores the impact of biopolymer-based organic nanofibers—specifically peptidomimetics formed through the self-assembly of L-valine and nicotinic acid (NA) (denoted as M6) on *Stevia rebaudiana* in vitro propagation and specialized metabolite production. The central hypothesis was that such nanofibers, particularly when used as hormone carriers, can beneficially influence plant morphology, physiology, and biochemistry, thereby promoting the synthesis of antioxidant compounds with therapeutic potential. **Methods:** The nanofibers were tested either alone (M6) or as carriers of the plant hormone indole-3-acetic acid (IAA) (M6+IAA), supplemented to the cultivation MS medium at variable concentrations (0, 1, 10, and 50 mg L^−1^). **Results:** The results revealed that treatment with 10 mg L^−1^ M6 significantly enhanced shoot growth parameters, including the highest fresh weight (0.249 g), mean shoot height (9.538 cm), shoot number (1.95), and micropropagation rate. Plants treated with M6 alone outperformed those treated with M6+IAA in terms of shoot growth, total soluble sugars, and steviol glycoside content. Conversely, M6+IAA treatment more effectively promoted root initiation, the increased accumulation of mono- and dicaffeoylquinic acids, and boosted antioxidant enzyme activity. **Conclusions:** These findings highlight the potential of organic nanofibers, both with and without hormone loading, as novel tools for optimizing micropropagation and metabolite enhancement in *Stevia rebaudiana*.

## 1. Introduction

*Stevia rebaudiana* Bertoni is a low-calorie sweetener with widespread use in the food and pharmaceutical industries [1]. Its economic and medicinal value highlight the need for raw material production to respond to the increasing demand. Vegetative propagation via stem cuttings is a common technique for *S. rebaudiana* reproduction, but this approach is not able to generate a large number of plants [2]. Furthermore, the low viability of seeds has hindered the conventional breeding of the species. Micropropagation is an efficient tool for producing multiple homogeneous plants and increasing the levels of specialized metabolites [3]. The efficiency of in vitro propagation is determined by a combination of many factors [4]. Among these, the type of explant plays a crucial role, as different tissues vary in their regenerative potential. Likewise, the composition of the culture medium, particularly the availability of macro- and micronutrients, strongly affects morphogenesis and growth. Plant growth regulators (PGRs), in terms of their type, concentration, and relative ratios, are especially critical for directing organogenesis or somatic embryogenesis. In addition, genetic variability among cultivars can significantly influence the response to in vitro culture conditions, often leading to differences in regeneration capacity, growth rates, and morphogenetic outcomes [4]. The rooting response of some genotypes of *S. rebaudiana* is low and is affected by the stem cutting type and the types of cytokinins and auxins used [5,6,7]. Enriching the culture medium with additives such as phytohormones, amino acids, vitamins, and nanoparticles is a widely adopted strategy in plant biotechnology. These supplements have been shown to accelerate plant growth, enhance antioxidant enzyme activity, and modulate the synthesis of a broad spectrum of specialized metabolites [8,9], making them promising candidates for enhancing the biomass production of economically important plant species and promoting the accumulation of valuable metabolites.

Amino acids, in particular, have demonstrated a positive effect on plant development in vitro [8,10]. Beyond serving as a nitrogen source, branched-chain amino acids (leucine, isoleucine, and valine) play an essential role in protein synthesis and structural integrity [11]. Notably, the accumulation of free amino acids such as valine, phenylalanine, glycine, and alanine has been associated with increased stevioside levels in *S. rebaudiana* L. callus cultures [8]. Moreover, the type and concentration of amino acids significantly influence somatic embryogenesis, as evidenced in strawberry and other plant species [10]. Among these, proline at a concentration of 100 mg L^−1^ has shown superior efficacy compared to glutamine and alanine in inducing and supporting the development of somatic embryos. In contrast, lower (50 mg L^−1^) and higher (200 mg L^−1^) concentrations of amino acids have proven ineffective in promoting somatic embryo formation and development.

Trigonelline (N-methylnicotinic acid) and nicotinic acid N-glucoside are nicotinic acid (NA) conjugates produced in higher plants from nicotinic acid species [12,13]. As a component of multiple enzymes that participate in several biochemical processes, such as the synthesis of protein and/or IAA oxidase, amylase, and proteinase, NA is essential for a wide range of physiological processes in all plant species [14]. Nicotinic acid also serves as a building block for specific specialized metabolites, including nicotine, anabasin, and ricinine, in a limited number of plant species [15,16]. In plant cells, pyridine nucleotides (nicotinamide adenine nucleotides—NAD and NADP) function as coenzymes and participate in a variety of redox processes, with the NADH/NAD^+^ and NADPH/NADP^+^ ratios in cells regulating the activities of many oxidoreductase reactions. The de novo and salvage pathways operate to synthesize pyridine nucleotides in plant cells. Many species utilize salvage mechanisms to reuse nicotinamide and NA, byproducts of NAD and NADP degradation, as substrates for the synthesis of pyridine nucleotides, known as the pyridine nucleotide cycle [17,18,19]. From this, it may be suggested that an exogenous supply of NA to the culture medium will be beneficial for plant in vitro propagation.

In addition to being essential for major physiological processes, from germination to growth, from flowering to fruit setting, and finally to abscission and senescence, plant growth regulators (PGRs) also help plants overcome stress. Indole-3-acetic acid (IAA) is an essential auxin produced by plants as it is crucial to many plant functions, including leaf formation, embryo development, root initiation and development, abscission (leaf falling), phototropism, geotropism, fruit development, etc. IAA promotes the growth of more root branches, root hairs, and root laterals, which increase the length of the roots and facilitate the uptake of nutrients from the environment [20]. IAA, at low concentrations and in combination with cytokinin, has been used in shoot multiplication media and to promote the micropropagation of *S. rebaudiana* in numerous protocols [21,22,23]. Its supplementation in the nutrient medium is essential for root induction, significantly enhancing both in vitro and ex vitro plantlet survival. Among the auxins tested, IAA was the most effective in promoting the formation and development of in vitro roots in *S. rebaudiana* [24,25].

The micro/nanoencapsulation of PGRs in polymer-based carriers is a suitable, cost-effective, and environmentally friendly alternative to traditional application and delivery methods, such as spraying [26]. The nanoparticles’ small size and high surface area have drawn the attention of many studies towards their characterization and formulation, as they offer improved applications [27]. They are useful in various industrial organic NPs that can function as nanocarriers for different nutrient elements or biologically active compounds. Establishing an effective interaction mechanism between NPs and PGRs, along with determining their optimal doses, is crucial for controlling their effects. As there are no studies in this subject, the use of nanosized organic peptidomimetics as phytohormone carriers could open new pathways in plant biotechnology.

In genotypes with poor rooting capabilities, auxin-loaded nanoparticles may improve rooting success with their unique physicochemical characteristics [28,29]. Zinc oxide nanoparticles loaded with IAA and IBA added to apple microcuttings in vitro resulted in 1.5 times higher in IBA-nZnO (56.3%) mean rooting percentage compared to IBA (38.0%) and 1.9 times higher in IAA-nZnO (34.6%) compared to IAA (17.8%) [9]. When plants are exposed to environmental stress, they generate endogenously reactive oxygen species (ROS) capable of oxidizing important biomolecules. Plants initiate their antioxidant defense system to neutralize the destructive actions of ROS. The enzyme antioxidant system that maintains a healthy redox state includes the following: superoxide dismutase (SOD), catalase (CAT), glutathione, glutathione peroxidases, and reductases. The vitamins and phenolic compounds serve as non-enzymatic antioxidants. The chlorogenic acids (CGAs) are part of the phenolic compounds, generated by the esterification of caffeic acids with quinic acid. CGAs donate hydrogen atoms to reduce free radicals and to inhibit oxidation reactions.

Given the limited studies in combining nanosized organic fiber with phytohormones, this combination represents a promising strategy worth further dose optimization. Recently, we explored self-assembling peptidomimetic-based organic nanofibers for delivering colloidal silver [30]. This compound possesses a bolaamphiphilic molecule, built by L-valine and nicotinic acid residues and was used as a carrier of silver. Our studies have been restricted to the study of this nanofiber alone to form organo- and hydrogels [31]. It is our first study where we used it as a carrier of another organic compound based on supramolecular nanostructures formation.

The objective of this study is based on the hypothesis that the application of M6 and M6+IAA will positively influence the physiology, biochemistry, and morphology of in vitro cultivated *S. rebaudiana* plantlets, thereby promoting the formation of pharmacologically valuable antioxidant compounds. To conduct this study, we utilized a peptidomimetic as an IAA carrier, formed by connecting valine and nicotinic acid fragments joined and duplicated with a diaminohexane spacer. This compound can produce nanofibrillar networks both in organic solution and in the absence of solvent. We hypothesize that valine-based peptidomimetics will help to stabilize and release IAA more slowly, resulting in more efficient delivery to the plant.

## 2. Materials and Methods

### 2.1. Chemicals and Reagents

Trifluoroacetic acid, decanoic acid, DIPEA [N,N-Diisopropylethylamine], and silver nitrate were purchased from Alfa Aesar (Haverhill, MA, USA). Boc-L-Asp(OBzl)-OH, TBTU [O-(Benzotriazol-1-yl)-N,N,N′,N′-tetramethyluronium tetrafluoroborate] were obtained from Iris Biotech (Marktredwitz, Germany). Dimethylformamide, citric acid, chloroform, ethyl acetate, hexane, NaHCO_3_, and methanol were purchased from Merck (Darmstadt, Germany). From Sigma-Aldrich (Jefferson, MO, USA), we obtained 5% Pd/C (palladium/carbon as a catalyst), nitroblue tetrazolium, riboflavin, and methionine. Murashige and Skoog nutrient medium and indole-3-acetic acid (IAA) were purchased from Duchefa Biochemie B.V, Haarlem, the Netherlands. Ascorbate, guaiacol, and 30% H_2_O_2_ were obtained from Merck (Darmstadt, Germany). Stevioside, rebaudioside A, chlorogenic acid (5-CQA), 3,5-dicaffeoylquinic acid (3,5-DCQA), 4,5-dicaffeoylquinic acid (4,5-DCQA), and quercetin 3-O-rhamnoside were purchased from Phytolab GmbH & Co. KG, Vestenbergsgreuth, Germany. All other chemicals were of analytical grade. Solvents used for HPLC analysis were of HPLC grade.

### 2.2. Chemical Nanofibers Synthesis

Compound M6 was synthetized using an already published procedure [31]. A solution of the compound M6 (80 mg) in 10 mL of 0.1 M HCl was prepared in a beaker. In another beaker, IAA (20 mg) was dissolved in 12 mL of 0.1 N NaOH. The careful mixing of both solutions resulted in the formation of a hydrogel. The molar ratio in the hydrogel between M6 and IAA was 3:1 (9:1 by mass). The obtained hydrogel was left to dry to obtain a dry mass, which was subsequently provided for analysis and for further biological testing. The gel network formed by M6 upon adding the base to the solution incorporated molecules of IAA, which are not visible by the microscope. A scanning electron microscope JEOL JSM 6390 (JEOL Ltd., Masashimmurayama, Tokyo, Japan) equipped with a digital camera was used to take microphotographs. The accelerating voltage was 10 keV in all cases. Before electron microscope observations, the xerogel samples were covered by a nanosized layer of Au for better conductivity.

### 2.3. Plant Material

Stem tips (2–3 cm) were excised from adult plants obtained from the ex situ collection of the Institute of Ornamental and Medicinal Plants, Sofia. The explants were surface-sterilized in 0.04% HgCl_2_ (*w*/*v*) for 20 min, rinsed three times with sterile distilled water and subsequently cultured aseptically in tubes (140 × 20 mm) containing half-strength MS medium supplemented with 0.5 mg L^−1^ indole-3-acetic acid (IAA).

The in vitro culture conditions followed the protocol of Zayova et al. [23]. Nodal stem explants with a single node (1–1.5 cm) were cultured on MS media containing M6 or M6+IAA at varied concentrations (1, 10, and 50 mg L^−1^). For the control, plantlets were grown on an MS nanoparticle-free medium. Forty stem explants were placed on each of the seven medium variants, and each treatment was repeated twice. The medium pH was adjusted to 5.7 ± 0.2 with 1 N NaOH or 1 N HCl prior to autoclaving (1.1 kg cm^−2^, 121 °C, 20 min). Cultures were maintained at 25 ± 1 °C under a 16 h light/8 h dark photoperiod, with irradiance of 40 μmol/m^2^/s provided by cool fluorescent tubes (36 W Philips). After four weeks of culture, the in vitro plantlets were removed from the culture vessels and collected for analysis.

### 2.4. Biometrics

Parameters assessed were shoots number per explant, shoot length, root length, and shoots and roots fresh weight. The potential micropropagation rate (MR) was then calculated using the equation below:

MR = SN × NN,

where SN is the number of newly produced shoots and NN is the number of new internodes formed per shoot.

### 2.5. Determination of Soluble Sugars

Total soluble sugars were quantified using the phenol–sulphuric acid method, following the procedure of Ashwell [32].

### 2.6. Determination of the Content of Stevioside and Rebaudioside A

Dried and finely ground aerial parts (50 mg) were extracted with distilled water (5 mL) at 40 °C in an ultrasonic bath for 30 min. After centrifugation and filtration, the filtrates were transferred to a volumetric flask, and the volume was made up to 5 mL with distilled water. One milliliter of each extract was passed through solid phase extraction (SPE) cartridges (Supeclean^TM^ LC-18, 500 mg, 3 mL, Supelco, Bellefonte, PA, USA) according to the procedure described in Bergs et al. [33]. A Shimadzu Nexera-I LC2040C 3D Plus liquid chromatograph equipped with a photodiode array detector (Shimadzu, Tokyo, Japan) was used for the quantitative determination of stevioside and rebaudioside A. The analysis was performed on an Inertsil NH2 column (150 × 4.0 mm, 3 µm) (GL Sciences, Torrance, CA, USA) at 210 nm with a mobile phase CH_3_CN/H_2_O (4:1, *v*/*v*) in an isocratic mode for 30 min. The temperature was maintained at 40 °C, and the flow rate was 0.8 mL min^−1^. The injection volume was 4 µL. The presence of stevioside and rebaudioside A was confirmed with the standards by comparing their retention times and UV spectra. Linear ranges, regression equations, correlation coefficients (R^2^), limits of detection (LOD), limits of quantification (LOQ), and calibration curves are given in the Supplementary Materials (Table S1, Figures S2 and S3). All experiments were performed in triplicate. The content of stevioside and rebaudioside A was expressed in mg per one gram of dry plant material (mg g^−1^ DW).

### 2.7. Determination of the Content of Mono- and Dicaffeoylquinic Acids and Quercitrin

Dried and finely ground aerial parts (150 mg) were extracted twice with methanol (5 mL) at 25 °C in an ultrasonic bath for 30 min. After centrifugation and filtration, the combined filtrates were transferred to a volumetric flask and the volume was adjusted to 10 mL with methanol. One milliliter of each extract was passed through solid phase extraction (SPE) cartridges (Chromabond^®^C18ec, 200 mg, 3 mL, Macherey-Nagel, GMBH&Co, KG, Düren, Germany) to remove chlorophylls. The same equipment as that described above (4.6.) was used for the quantitative determination of caffeoylquinic acids and quercitrin. The analysis was performed on a Force C18 column (150 × 4.6 mm, 3 µm) (Restek, Bellefonte, PA, USA) at a temperature of 40 °C, a flow rate of 0.6 mL min^−1^, and an injection volume of 2 µL. The elution was carried out with 0.1% (*v*/*v*) HCOOH in H_2_O (A) and 0.1% (*v*/*v*) HCOOH in CH_3_OH (B) in gradient mode as described previously in Sichanova et al. [34]. The runs were monitored at 320 nm (caffeoylquinic acids) and 350 nm (quercitrin). The presence of mono- and dicaffeoylquinic acids and quercitrin was confirmed with the standards by comparing their retention times and UV spectra. Linear ranges, regression equations, correlation coefficients (R^2^), limits of detection (LOD), limits of quantification (LOQ), and calibration curves are given in the Supplementary Materials (Table S2, Figures S4 and S5). The amounts of neochlorogenic (3-CQA) and cryptochlorogenic (4-CQA) were estimated from peak areas as equivalents of chlorogenic acid (5-CQA). All experiments were performed in triplicate. The mono- and dicaffeoylquinic acids and quercitrin content were expressed in mg per one gram of dry plant material (mg g^−1^ DW).

### 2.8. Antioxidant Activity

For the enzyme antioxidant activity (superoxide dismutase (SOD), catalase (CAT), ascorbate peroxidase (APX), and guaiacol peroxidase (GPO)), the extraction was carried out following the procedure of Hristozkova et al. [35]. Total SOD (EC 1.15.1.1) activity [36], CAT (EC 1.11.1.6) activity [37], APX (EC 1.11.1.1) activity [38], and GPO (EC 1.11.1.7) activity [39] were determined spectrophotometrically on UV/VIS spectrophotometer (Shimadzu UV-1601, Tokyo, Japan). The soluble protein content was determined by using bovine serum albumin as a standard [40].

### 2.9. Stress Markers Estimation

Fresh leaf material (300 mg) was homogenized with 0.1% (*w*/*v*) trichloroacetic acid for the determination of the content of hydrogen peroxide (H_2_O_2_), malondialdehyde (MDA), proline, and free thiol group-containing compounds (SH-). According to the protocol described by Alexieva et al. [41], the hydrogen peroxide content was measured spectrophotometrically. Malondialdehyde, free proline, and free thiol contents were quantified following Kramer et al. [42], Bates et al. [43], and Ellman [44], respectively. MDA was measured as a thiobarbituric acid–reagent product using an extinction coefficient of 155 mM^−1^ cm^−1^. Free proline absorbance was read at 520 nm after reaction with acidic ninhydrin, forming a red-colored complex. Free thiol groups were quantified at 412 nm after incubating 40 µL of supernatant with 150 µL of Ellman’s reagent for 10 min at room temperature.

### 2.10. Statistical Analysis

Principal component analysis (PCA) was performed on the whole set of data for the content of individual compounds by using Past 4.06b software (stevioside; rebaudioside A; 3-, 4-, and 5-caffeoylquinic; and 3,5- and 4,5-dicaffeoylquinic acids and quercitrin) (https://www.nhm.uio.no/english/research/resources/past/ (accessed on 16 May 2025)). The data on biometric parameters, soluble sugars, antioxidant enzyme activities, and stress markers were analyzed using a one-way analysis of variance (ANOVA). Significant differences between groups were determined using Fisher’s least significant difference (LSD) test at a 5% significance level, employing the Statgraphics Plus software (version 5.1 for Windows). Results are presented as mean values ± standard error (SE).

## 3. Results

**Figure 1:**
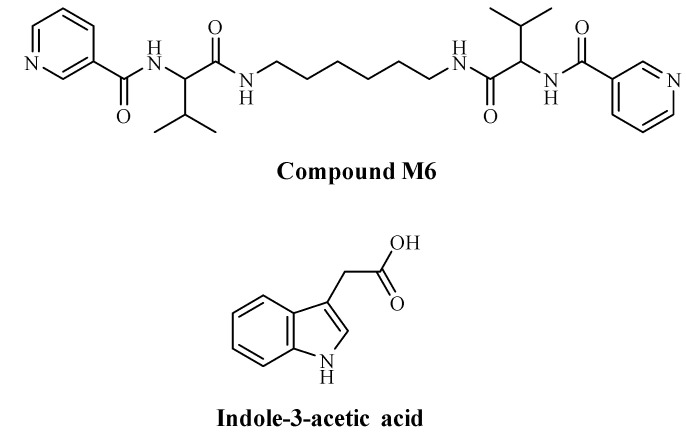
Structures of compound M6 and indole-3-acetic acid (IAA).

The organic compound M6 (Figure 1) was synthesized by applying previously described procedures [31]. In this work, we decided to use indole acetic acid (IAA), which belongs to the auxin phytohormone family. IAA controls important physiological processes in plants, and it is a heterocyclic compound comprising a carboxymethyl group of acetic acid moiety and an indole aromatic residue (Figure 1). For this reason, we chose the compound M6, which also includes an aromatic system—two pyridine rings—so to induce π-π interactions responsible for the intermolecular interactions. Another possible link is between the carboxylic group of IAA and pyridine’s nitrogen, where the transfer of a proton, followed by electrostatic interactions, can happen.

**Figure 2:**
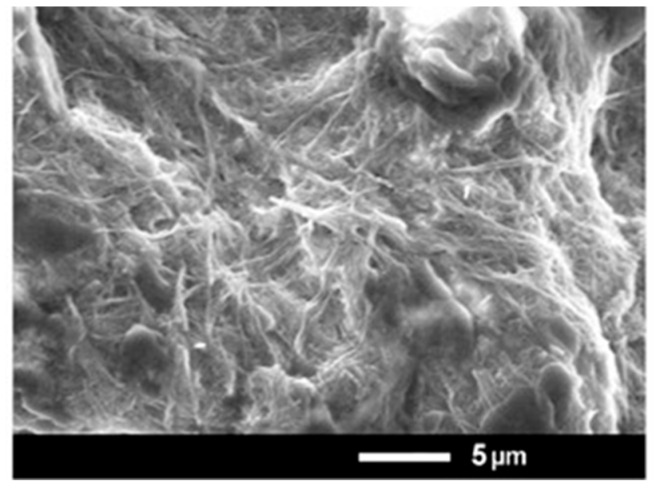
SEM microphotographs of the xerogel obtained from compound M6.

Nanofibers constructed by M6 and IAA have been formed through the primary formation of a hydrogel. This gel was a result of a self-assembly process where molecules interact with each other through physical forces, among which are electrostatic ones and π-π stacking. Most probably, the complex formation between M6 and IAA is due to these noncovalent bonds and interactions. Dried hydrogel (xerogel) was examined by an electron microscope, and nanofibers with diameters in the range of nanometers were well visible (Figure 2). The molar ratio in the hydrogel formed by M6 and IAA is 3:1, corresponding to a mass ratio of 9:1. Thus, the concentrations of IAA within the M6+IAA complex corresponded to 0.1, 1.0, and 5.0 mg L^−1^.

The complexes formed by the two compounds were studied using a UV–Vis spectrophotometer. For this purpose, a gel was prepared with the compounds in amounts 40 times smaller than those described above. The xerogel obtained after drying was dissolved in 2 mL of water, and 133 µL of it was dissolved in 4 mL of water. Samples were prepared in the same way for the pure compounds separately.

The UV absorption spectrum of indole-3-acetic acid (IAA) exhibited a major peak at 220 nm and three overlapping peaks around 274, 282, and 288 nm with low intensity, that was due to the indole aromatic structure. M6 contains a chromophore nicotinoyl residue and shows a main peak at 262 nm (Figure S1). The complex between both compounds showed two main peaks, one of which was the same as the M6 peak, and the second was analogous to the IAA main peak but shifted to 215 nm.

Furthermore, although the low intensity of the IAA peaks in the 274–288 nm region disappeared at the complex peak, this may be regarded as shifting to the peak at 262 nm due to pi–pi stacking with the pyridine rings of M6. From this study, we can conclude that M6 does not change its absorption in the complex, most likely because it exists as supramolecular complexes in both cases—without IAA and loaded with IAA—whereas IAA without M6 is a molecular solution. In the presence of M6, however, it participates as part of the supramolecular structure, causing a shift in absorption to the lower wavelength region (blue shift). This shift may result in its absorbance in the 274–288 nm region moving to around 260 nm.

**Figure 3:**
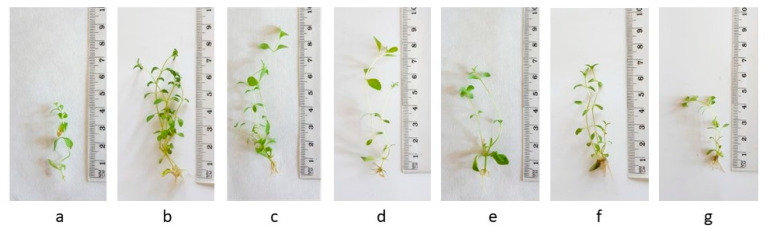
*Stevia rebaudiana* plantlets cultured on MS medium, supplemented with various concentrations (0 (**a**), 1 (**b**), 10 (**c**), and 50 (**d**) mg L^−1^) of amino acid nanofibers M6, and on MS medium supplemented with various concentrations (1 (**e**), 10 (**f**), and 50 (**g**) mg L^−1^) of M6+IAA.

The effects of adding M6 and M6+IAA to the MS nutrient medium on the growth and development of *S. rebaudiana* were studied. All the treatments showed good responses for both shoot and root induction (Table 1, Figure 3). The results obtained after 4 weeks of cultivation using organic nanoparticle M6 showed that all tested morphogenic parameters (the number of shoots formed per explant, shoot height, newly formed nodes, and root efficiency) increased compared to the control untreated plantlets.

Optimal results were achieved in plants cultured on 10 mg L^−1^ M6, which had the maximum shoot elongation (9.5 cm), the highest multiplication rate (9.28), and the highest rooting frequency (85%), which correlated with the highest FW (0.249). The multiplication rate also increased when M6+IAA was applied in the MS nutrient media (6.29–7.22), compared to the control (2.35). However, the shoot elongation was reduced. High rooting efficiency (100%) was observed after treatments with 1 mg L^−1^ M6+IAA and 10 mg L^−1^ M6+IAA. Among the tested concentrations of M6+IAA, 10 mg L^−1^ M6+IAA exhibited the best results regarding measured shoot number, node number and multiplication rate.

The addition of the organic nanofiber M6 to the nutrient medium positively affected sugar production in *S. rebaudiana* plantlets compared to control plantlets (Table 2). The presence of M6+IAA in the cultural medium stimulated soluble sugar synthesis only at a concentration of 1 mg L^−1^, while the levels of soluble sugars were significantly reduced at 10 and 50 mg L^−1^.

**Figure 4:**
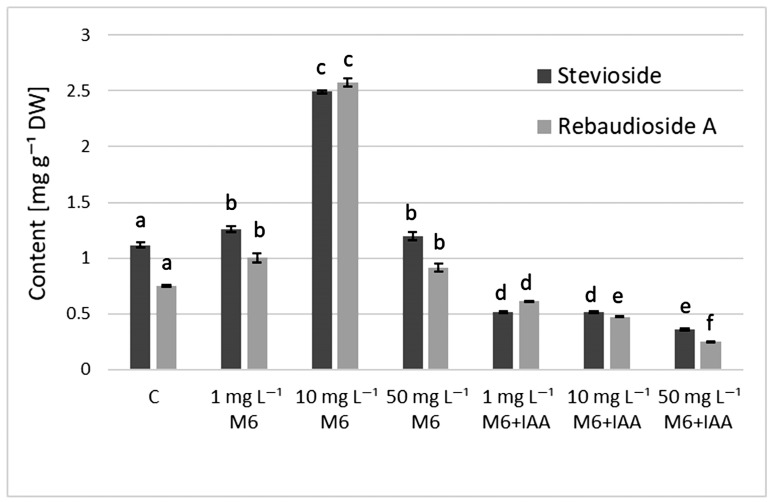
The content of steviol glycosides in *S. rebaudiana* plantlets cultured on MS medium, supplemented with 0, 1, 10, or 50 mg L^−1^ of M6 or M6+IAA. Values are means ± SD (from three measurements). Values with different letters for each bar are significantly different (*p* ≤ 0.05) according to Tukey’s test.

The results from the quantitative determination of stevioside and rebaudioside A in the *S. rebaudiana* plantlets (Figure 4) showed that the addition of organic nanoparticle M6 to the culture medium stimulated the production of steviol glycosides (SGs) in *S. rebaudiana* plantlets and the highest content of both compounds was recorded at 10 mg L^−1^ M6 (2.493 and 2.578 mg g^−1^ DW). Furthermore, compared to control plants and the ones grown in MS medium containing only M6, the stevioside and rebaudioside A content decreased when M6+IAA carrier was added to the MS medium.

The quantitative analysis of mono- (CQA) and dicaffeoylquinic (DCQA) acids, along with quercetin-3-O-rhamnoside (quercitrin), in *S. rebaudiana* plantlets is summarized in Table 3. Chlorogenic acid (5-CQA) was the dominant component among the monocaffeoyl esters of quinic acid in all samples, detected at levels ranging from 0.289 to 3.066 mg g^−1^ DW. In contrast, neochlorogenic (3-CQA) and cryptochlorogenic (4-CQA) acids occurred at much lower levels, ranging from 0 to 0.071 mg g^−1^ DW and 0.027 to 0.152 mg g^−1^ DW, respectively. Further, 3,5-DCQA (0.970–5.735 mg g^−1^ DW) was the main dicaffeoylquinic acid, while the content of 4,5-DCQA was considerably low (0.223–0.944 mg g^−1^ DW). The total amount of DCQAs surpassed that of CQAs in all samples (Table 3), and the DCQA/CQA ratio ranged from 1.5 (C) to 3.3 (50 mg L^−1^ M6). The highest total content of CQAs and DCQAs was observed in the plantlets propagated in vitro on MS medium containing 10 mg L^−1^ M6+IAA. Moreover, it was noted that the presence of M6+IAA in the MS medium stimulated the production of all individual CQAs and DCQAs, except 3-CQA. Additionally, the levels of CQAs and DCQAs decreased with increasing concentrations of M6 and M6+IAA up to 50 mg L^−1^.

The highest content of quercitrin (Table 3) was found in the plantlets propagated on MS medium containing 1 mg L^−1^ M6+IAA (0.145 mg g^−1^ DW). As can be seen, the addition of M6+IAA to the culture medium led to a larger amount of quercitrin than the plantlets grown on MS medium with M6 alone. Increasing the concentration of M6 and M6+IAA in the culture medium resulted in a decrease in quercitrin content.

**Figure 5:**
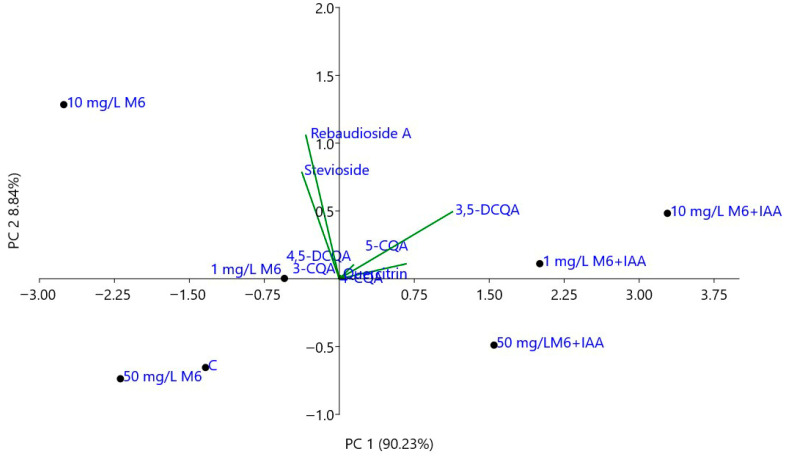
Biplot from PCA performed on the mean content of the individual compounds in *S. rebaudiana* plantlets in vitro propagated on MS and MS media supplemented with different concentrations of M6 and M6+IAA.

The principal component analysis (PCA) of individual compounds, including stevioside; rebaudioside A; 3-, 4- and 5-caffeoylquinic; 3,5- and 4,5-dicaffeoylquinic acids; and quercitrin, was performed to assess the variation in the chemical composition of *S. rebaudiana* plantlets propagated in vitro on MS medium and MS medium supplemented with different concentrations of M6 and M6+IAA. As can be seen in Figure 5, the first two principal axes accounted for 99.07% of the total variations. PC1 (90.23% of total variations) was positively related to 3,5-DCQA, 5-CQA, 3-CQA, 4,5-DCQA, and 4-CQA and negatively related to stevioside and rebaudioside A. The plantlets propagated in vitro on MS medium, supplemented with various concentrations (1, 10, and 50 mg L^−1^) of M6+IAA, were clearly differentiated from the control and those propagated on MS medium supplemented with various concentrations of M6. Further, PC2 (8.84% of total variations) distinguished the plantlets in vitro propagated on MS medium (C) and those propagated on MS media supplemented with 50 mg L^−1^ of M6 and M6+IAA from the others. Thus, the plantlets grown on MS supplemented with 50 mg L^−1^ of M6+IAA, which is characterized by the lowest content of stevioside and rebaudioside A, are located in the positive side of PC2 and the negative side of PC1. The plantlets grown on MS supplemented with 50 mg L^−1^ of M6 and those propagated on MS medium (C) are positioned on the negative side of both PC1 and PC2 and are characterized by the lowest content of caffeoylquinic acids and a moderate content of steviol glycosides.

**Figure 6:**
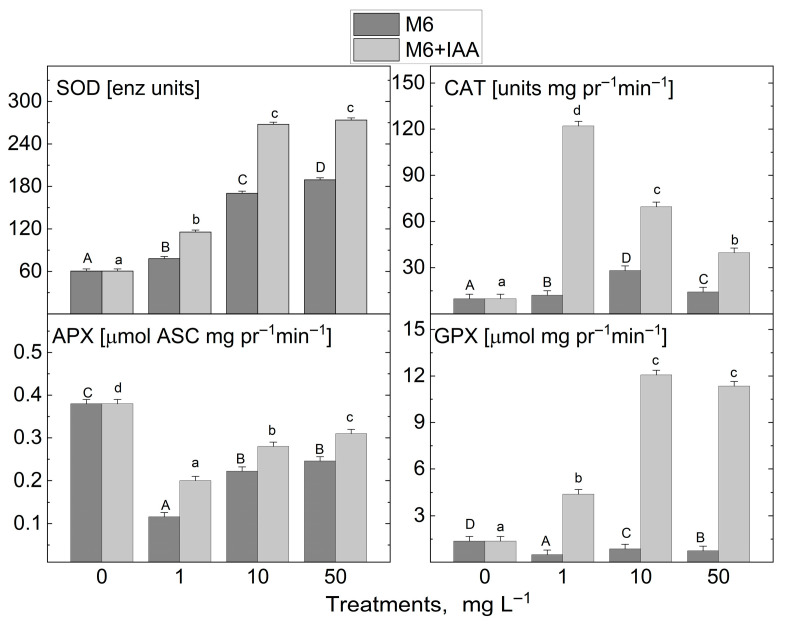
Activity levels of antioxidant enzymes superoxide dismutase (SOD), catalase (CAT), guaiacol peroxidase (GPX), and ascorbate peroxidase (APX) in *S. rebaudiana* plantlets cultured on MS medium, supplemented with 0, 1, 10, or 50 mg L^−1^ of M6 or M6+IAA. pr—protein; values are means ± SE, *n* = 20; different letters indicate significant differences (Fisher LSD test, *p* ≤ 0.05) following one-way ANOVA analysis. Letters “a”/“A” denote the lowest values, with subsequent letters assigned to higher values. The statistical analyses were conducted separately for M6 (uppercase) and M6+IAA (lowercase).

The addition of the M6 to the MS nutrient medium at three tested concentrations (1, 10, and 50 mg L^−1^) resulted in an increase solely in the activity of the enzyme that captures the reactive oxygen radical and converts it into hydrogen peroxide, SOD, and the enzyme that neutralizes H_2_O_2_ to water, CAT, in the micropropagated *S. rebaudiana* plantlets compared to the untreated control plants (Figure 6). The activity of the other investigated antioxidant enzymes, APX and GPX, was reduced. After the treatment with M6+IAA, an even greater increase in SOD and CAT activity was observed, and GPX activity also increased compared to the plants cultivated on an MS nutrient medium devoid of nanofibers. Only the APX activity was reduced compared to the control under all treatments. SOD activity rose with increasing concentrations of M6 and M6+IAA. The highest increase in CAT activity was recorded when plants were cultivated at 1 mg L^−1^ M6+IAA, while a reduction in activity was measured with rising nanofiber concentrations. However, CAT activity levels remained higher than those of the control plants. With increasing concentrations of M6 and M6+IAA, an inverse trend in APX activity was recorded compared to that of CAT. The lowest CAT activity was observed at 1 mg L^−1^ M6 and M6+IAA, but with increasing the concentration to 50 mg L^−1^, it rose but remained lower than that of untreated plants.

**Figure 7:**
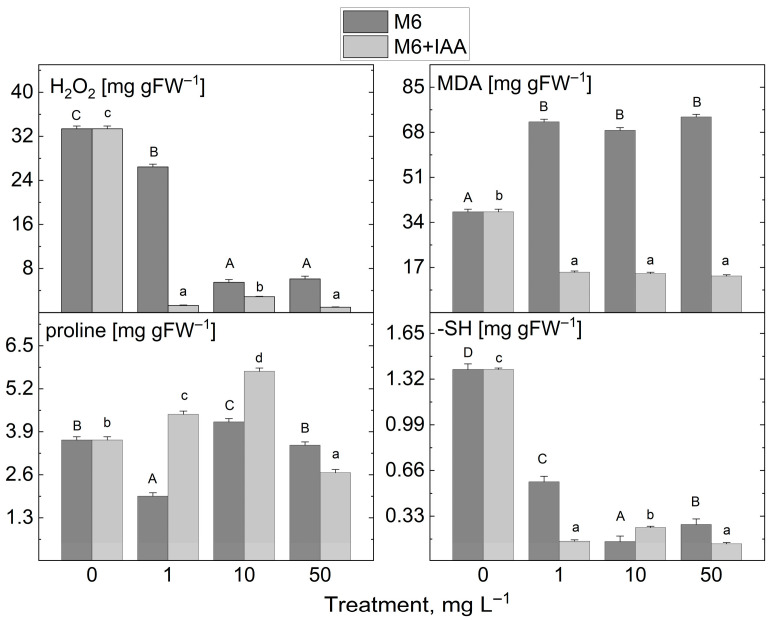
The levels of stress markers (H_2_O_2_, MDA, proline, and -SH groups) in *S. rebaudiana* plantlets cultured on MS medium, supplemented with 0, 1, 10, or 50 mg L^−1^ of M6 or M6+IAA. pr—protein; values are means ± SE, *n* = 20; different letters indicate significant differences (Fisher LSD test, *p* ≤ 0.05) following one-way ANOVA analysis. Letters “a”/“A” denote the lowest values, with subsequent letters assigned to higher values. The statistical analyses were conducted separately for M6 (uppercase) and M6+IAA (lowercase).

The addition of 1 mg L^−1^ M6 to the MS nutrient medium during in vitro propagation led to a slight decrease in H_2_O_2_ content compared to control plants (Figure 7). However, the application of the other M6 concentrations studied, along with the M6+IAA addition at all tested concentrations, drastically reduced H_2_O_2_ levels in *S. rebaudiana* plantlets. A similar trend was observed for MDA after treatment with M6+IAA across all tested concentrations. A significant increase in MDA levels was noted in extracts from plantlets treated with the three concentrations of M6. Treatment with the two types of nanofibers at the three investigated concentrations resulted in a decrease in sulfhydryl group levels. Monitoring the changes in proline content following M6 and M6+IAA treatment revealed two distinct trends: an increase when plants were treated with 1 and 10 mg L^−1^ M6+IAA, and a reduction solely at 50 mg L^−1^ M6+IAA, a decrease at 1 and 50 mg L^−1^ M6, and an increase at 10 mg L^−1^ M6.

## 4. Discussion

The main biometric parameters of 30-day-old *S. rebaudiana* micro plantlets during in vitro propagation were determined. The analysis of the results revealed that the synthesized organic nanofibers either applied alone (M6) or as an IAA carrier (M6+IAA) (Figure 1), when added to the MS medium during direct organogenesis, positively influenced the growth parameters of *S. rebaudiana*, including fresh biomass accumulation, shoot height, the number of shoots per explant, the number of nodes per shoot, and the potential micropropagation rate. Additionally, the percentage of root formation was higher in plants treated with nanofibers than in the controls. When *S. rebaudiana* was treated with IAA added to the MS nutrient medium at different concentrations, both shoot height and the number of shoots per explant, as well as the number and length of roots, were highest only at 1 mg L^−1^ IAA compared to the control plants [25]. An increase in IAA concentration led to a reduction in plantlet growth. The reported results for a 1 mg L^−1^ IAA treatment [25] are nearly identical to those obtained when M6+IAA was added to the culture medium. M6 treatment improved shoot growth characteristics more than M6+IAA, while the percentage of shoots that formed roots was higher after M6+IAA treatment. The studied nanoparticle M6 is a compound with a nanofiber structure, which possesses a bolaamphiphile structure composed of two valines and a nicotinic acid linked together through fragments and doubled by a diamino hexane spacer (Figure 1) [30]. The auxin IAA is incorporated into the micelles formed by M6, which then arrange in formation or are integrated on the surfaces of the nanofibers formed by M6. It could be suggested that the changes in the plantlet growth and antioxidant activity could be influenced by the combined action of the organic nanofiber constituents (amino acid valine, nicotinic acid) and the auxin IAA.

IAA is a key inducer of root initiation [45]. It improved stevia growth by promoting root formation (increasing root length and root number), plant height, leaf number, stomatal density, and chlorophyll content [46]. Furthermore, auxin is suggested to promote starch breakdown into soluble sugars, which are then transported to the cutting base, inducing root regeneration [47]. This likely explains the lower sugar content observed in the above-ground parts of stevia treated with M6+IAA compared to those treated with M6 alone. Javed et al. [48] found a positive effect of IAA on stevioside and rebaudioside A content in in vitro cultivated *S. rebaudiana*. Surprisingly, a reduction in the stevioside and rebaudioside A content of the *S. rebaudiana* plantlets was measured when plantlets were treated with M6+IAA at three tested concentrations, compared to the M6-treated plantlets. Increased SGs levels were recorded only in the variants treated with M6 compared to controls. The same results were obtained for parameters characterizing plant growth. A decline was measured in the plantlets’ fresh weight and height, as well as potential micropropagated rates, when *S. rebaudiana* plantlets were cultivated on MS enriched with M6+IAA, compared with the treatment using M6 alone. IAA modulates several metabolic processes, such as cell division, differentiation, and elongation. IAA may cause plants to prioritize growth and development over the synthesis of specialized metabolites, like SGs. It could be suggested that reduced SGs accumulation may result from this change. Increased root and shoot elongation have been linked to IAA in tissue culture systems [49]. Nevertheless, such conditions often lead to reduced differentiation and the synthesis of specialized metabolites, such as SGs. This implies that IAA may simultaneously inhibit SGs biosynthesis while promoting specific growth aspects. The results obtained could be due to the combined effect of M6 and IAA.

The higher activity of antioxidant enzymes in the plants treated with M6+IAA, compared to those treated with M6 alone, indicates that IAA has an inducing effect on the studied enzymes. Increased SOD, CAT, and GPO activity levels have been reported in maize seedlings after treatment with IAA [50]. The positive role of IAA in alleviating oxidative stress has been documented in various plant species [51,52]. Significantly, an increase in total phenolic content when IAA was applied during in vitro *S. rebaudiana* propagation was also reported [53]. The treatment with M6 and M6+IAA also induced higher caffeoylquinic acid accumulation in micropropagated *S. rebaudiana* plantlets. The highest levels of all five phenolic compounds were obtained for 3-CQA and 3,5-DCQA. Similar results were reported when *S. rebaudiana* was propagated from seed and in vitro in a cultivation medium containing kinetin [54,55]. Ma et al. [52] demonstrated that the exogenous application of IAA can alleviate alkaline-stress-induced growth inhibition in rice plants by modulating the reactive oxygen species scavenging system. The authors reported higher activities of the antioxidant enzymes SOD, CAT, and GPO, accompanied by lower levels of stress markers H_2_O_2_ and MDA, after treatment with IAA. IAA treatment of Cu-stressed spinach seedlings was shown to increase SOD, APX, and glutathione reductase (GR) activities as well as proline content. At the same time, the MDA level decreased, thus alleviating the negative effects of Cu [53]. In the present study, M6+IAA application also resulted in lower levels of H_2_O_2_ and MDA, as well as higher proline content, compared to both the control and M6-treated plantlets.

Many species utilize salvage pathways to reuse nicotinamide and nicotinic acid (NA), byproducts of NAD and NADP breakdown, for pyridine nucleotide synthesis [56]. Nicotinic acid acts as a precursor that produces pyridine nucleotides (mainly NAD and NADP—coenzymes vital for various metabolic processes and macro- and micronutrient utilization) exclusively via the salvage biosynthetic pathway, not through de novo synthesis. Studies have shown that after 18 h of incubation with [^14^C] nicotinic acid in the suspension-cultured cells of white spruce (*Picea glauca*), 32–58% of the compounds’ total radioactivity was detected in pyridine nucleotides (mostly NAD and NADP) and trigonelline [57]. The overexpression of the NIC3 gene, which encodes a key enzyme responsible for converting nicotinamide (NAM) to nicotinic acid (NA) within the NAD biosynthesis salvage pathway, leads to elevated NA levels in plants. This metabolic enhancement strengthens drought stress tolerance and promotes overall plant growth by activating a suite of genes linked to both growth regulation and stress resilience [58]. Interestingly, exogenous supplementation with NA mimicked the effects of NIC3 overexpression, resulting in similar physiological and molecular responses, including improved drought tolerance and enhanced growth capacity. These findings suggest that both genetic manipulation of NIC3 and external NA application converge on a common mechanism, highlighting NA as a critical metabolite in coordinating stress adaptation and growth regulation. Similar results have been reported concerning the beneficial effects of exogenously applied NA as a foliar spray on wheat’s drought tolerance, encouraging root growth (length, fresh and dry weights, surface area, diameter) and shoot growth (length, fresh and dry weights). Furthermore, NA effectively activates the antioxidant system in wheat, as evidenced by significantly elevated levels of APX, CAT, GPX, and SOD [59]. Additionally, it has been observed that the foliar application of nicotinic acid significantly boosts leaf area, shoot and root fresh weight, photosynthetic pigments, photosynthetic parameters, and total nitrogen content in fenugreek (*Trigonella foenum-graecum* L.) [60]. The increased activity of SOD and CAT in *S. rebaudiana* plantlets, alongside reduced levels of H_2_O_2_ and MDA, under the influence of the tested nanofibers M6 and M6+IAA, is likely attributable to the pyridine structure of nicotinic acid at the ends of the M6 molecule. Moreover, the supplementation with M6 and M6+IAA led to a notable increase in osmoregulatory substances such as proline.

When Brassicaceae plants are subjected to salt stress to mitigate its harmful effects, it secretes low molecular weight peptides [61]. In our experiments, we added peptidomimetics (M6 and M6+IAA) to the MS nutrient medium, which are small peptide-based molecules (short-chain amino acid sequences) that mimic the physicochemical properties and biological activity of antimicrobial peptides. These help plants cope with in vitro reproductive stress, resulting in positive effects on growth and increased antioxidant activity. The amino acids provide building blocks for protein synthesis, essential for cell division and elongation [62] throughout the plant. Conversely, valine, a key component of the M6 molecule, likely also influences growth and antioxidant activity. Similar results were reported by Wu et al. [63], who measured the valine–threonine–isoleucine–aspartic acid (VTID) accumulation in salt-stressed maize seedlings. According to the authors, these amino acids assist maize in adapting to salt stress. By adding this set of acids exogenously to salt-stressed maize seedlings, the authors observed a significant increase in plantlet height, antioxidant enzymes (especially SOD and CAT), and osmoregulatory substances such as proline, alongside a decrease in MDA levels. Amino acids serve not only as a nitrogen source that plants can directly absorb but also as the primary transport form of organic nitrogen in plants. Furthermore, the essential non-polar amino acid valine is one of the amino acids included in the important so-called branched-chain amino acids (valine, leucine, and isoleucine), which are building blocks of plant proteins [64]. It has been established that valine partially alleviated the growth inhibition of soybean cells (*Glycine max* L. var. *Amsoy* 71) in suspension culture caused by sulfonylurea herbicides, which block valine and leucine synthesis [65].

If nanoparticles (NPs) are not used in optimal amounts, they act like other stress agents [66]. Due to their potentially harmful nature, NPs have been shown to negatively affect plants under certain conditions, such as concentration, size, nature, shape of nanoparticles, plant species, the stage of plant development, and environmental factors. As a result, the research findings on NPs have not always been positive. The most common plant response to NPs toxicity is the generation of specialized metabolites and the activation of antioxidant mechanisms [66]. The results of our study showed that applying M6+IAA at concentrations of 1, 10, and 50 mg L^−1^ during *S. rebaudiana* micropropagation increases the activities of SOD, CAT, and GPX, as well as the levels of key metabolites with antioxidant potential such as quercetin 3-O-rhamnoside, CQAs, and DCQAs. The highest growth parameters for *S. rebaudiana* plantlets were observed at 1 and 10 mg L^−1^ M6 and M6+IAA. Increasing the concentration to 50 mg L^−1^ caused a decline in growth indicators. Adding 100 mg L^−1^ to the MS nutrient medium completely inhibited the development of stem explants (Figure S7). Radić et al. [53] reported a significant increase in phenols and flavonoids in stevia leaves cultivated in vitro on medium supplemented with 0.5 and 1.0 mg L^−1^ IAA. However, at a higher auxin concentration (1.5 mg L^−1^), their accumulation was reduced. Rokosa and Kulpa [25] observed enhanced growth in stevia treated with 1 mg L^−1^ IAA, whereas higher concentrations (2, 4, and 8 mg L^−1^) led to a reduction in fresh weight, stem length, and root number.

## 5. Conclusions

Our current study demonstrates that M6 and M6+IAA nanofibers hold significant potential for application in stevia tissue culture technologies. They positively affect stevia growth across all tested concentrations and, at appropriate levels, enhance the synthesis of specialized metabolites. Treating the plantlets with M6 influenced their metabolism towards synthesizing steviol glycosides, particularly stevioside and rebaudioside A, with optimal results observed at 1 mg L^−1^. Conversely, the addition of M6+IAA to the MS nutrient medium guided the metabolism towards producing mono- and dicaffeoylquinic acids, reaching their peak levels at 10 mg L^−1^. However, when M6 and M6+IAA were added to the culture medium at the highest tested concentration of 50 mg L^−1^, they had a negative effect, leading to a reduced content of steviol glycosides and caffeoylquinic acids.

This study highlights the potential of M6 and M6+IAA nanofibers as innovative metabolic modulators in *Stevia rebaudiana* tissue culture and their possible application in guiding the synthesis of target metabolites. The controlled application of nanofibers could be a valuable strategy for optimizing stevia propagation and metabolite production. Future studies should focus on unraveling the underlying molecular mechanisms governing these shifts in metabolism. Additional research is required to determine what happens to the nanofiber–auxin complex in soil, encompassing its stability, mobility, and degradation over time.

## Figures and Tables

**Table 1:** Morphological parameters of *S. rebaudiana* plantlets cultured on Murashige and Skoog (MS) medium, supplemented with various concentrations of M6 and M6+IAA.

	Shoot FW, g	Shoot Height, cm	Shoot Numbers Explant^−1^	Node Numbers Explant^−1^	MR	Rooting %
C	0.12 ± 0.01 ^a^	4.53 ± 0.223 ^a^	1.02 ± 0.05 ^a^	2.31 ± 0.12 ^a^	2.35 ± 0.12 ^a^	0.03
M6						
1 mg L^−1^	0.23 ± 0.01 ^c^	7.62 ± 0.38 ^c^	1.75 ± 0.09 ^c^	4.26 ± 0.21 ^b^	7.46 ± 0.37 ^cd^	65
10 mg L^−1^	0.25 ± 0.01 ^d^	9.54 ± 0.48 ^d^	1.95 ± 0.10 ^d^	4.76 ± 0.24 ^b^	9.28 ± 0.46 ^e^	85
50 mg L^−1^	0.21 ± 0.01 ^c^	7.19 ± 0.36 ^c^	1.65 ± 0.08 ^bc^	4.80 ± 0.24 ^b^	7.92 ± 0.40 ^d^	75
M6+IAA						
1 mg L^−1^	0.19 ± 0.01 ^b^	5.41 ± 0.27 ^b^	1.70 ± 0.09 ^c^	3.88 ± 0.19 ^b^	6.59 ± 0.33 ^b^	100
10 mg L^−1^	0.19 ± 0.01 ^b^	5.71 ± 0.29 ^b^	1.75 ± 0.09 ^c^	4.13 ± 0.21 ^b^	7.22 ± 0.36 ^c^	100
50 mg L^−1^	0.18 ± 0.01 ^b^	4.71 ± 0.24 ^a^	1.55 ± 0.08 ^b^	4.06 ± 0.20 ^b^	6.29 ± 0.32 ^b^	85
LSD	0.02	0.58	0.14	1.02	0.61	

MR—micropropagation rate; values are means ± SE, *n* = 20; different letters indicate significant differences assessed by the Fisher LSD test (*p* ≤ 0.05) after performing one-way ANOVA analysis. We used the letter “a” for the lowest data value and proceeded to the next letters in the alphabet for higher data values.

**Table 2:** The content of sugars in *S. rebaudiana* plantlets.

	Sugars [mg g^−1^ FW]
C	10.25 ± 0.51 ^c^
M6	
1 mg L^−1^	14.22 ± 0.71 ^e^
10 mg L^−1^	13.78 ± 0.69 ^e^
50 mg L^−1^	13.48 ± 0.67 ^e^
M6+IAA	
1 mg L^−1^	12.28 ± 0.61 ^d^
10 mg L^−1^	8.14 ± 0.41 ^b^
50 mg L^−1^	3.27 ± 0.16 ^a^
LSD	1.00

Values for sugars are means ± SE, *n* = 20; different letters indicate significant differences assessed by the Fisher LSD test (*p* ≤ 0.05) after performing one-way ANOVA analysis. We used the letter “a” for the lowest data value and proceeded to the next letters in the alphabet for higher data values.

**Table 3:** The content of caffeoylquinic acids and quercitrin in *S. rebaudiana* plantlets (mg g^−1^ DW).

	3-CQA	5-CQA	4-CQA	3,5-DCQA	4,5-DCQA	Quercitrin	CQAs	DCQAs
C	0.071 ± 0.004 ^a^	1.086 ± 0.042 ^a^	0.152 ± 0.016 ^a^	1.459 ± 0.027 ^a^	0.453 ± 0.013 ^a^	0.086 ± 0.004 ^a^	1.309 ± 0.063 ^a^	1.912 ± 0.039 ^a^
M6								
1 mg L^−1^	0.062 ± 0.003 ^b^	1.092 ± 0.014 ^a^	0.063 ± 0.003 ^b^	2.562 ± 0.050 ^b^	0.476 ± 0.005 ^b^	0.094 ± 0.001 ^b^	1.217 ± 0.009 ^a^	3.038 ± 0.054 ^b^
10 mg L^−1^	0.054 ± 0.001 ^c^	0.424 ± 0.008 ^b^	0.079 ± 0.002 ^c^	1.090 ± 0.010 ^c^	0.317 ± 0.007 ^c^	0.072 ± 0.002 ^c^	0.494 ± 0.011 ^b^	1.339 ± 0.017 ^c^
50 mg L^−1^	0.030 ± 0.001 ^d^	0.289 ± 0.007 ^c^	0.043 ± 0.003 ^d^	0.970 ± 0.008 ^d^	0.223 ± 0.006 ^d^	0.048 ± 0.001 ^d^	0.362 ± 0.010 ^c^	1.193 ± 0.014 ^d^
M6+IAA								
1 mg L^−1^	0.042 ± 0.004 ^e^	2.557 ± 0.045 ^d^	0.144 ± 0.009 ^a^	4.467 ± 0.024 ^e^	0.944 ± 0.036 ^e^	0.145 ± 0.003 ^e^	2.743 ± 0.058 ^d^	5.412 ± 0.060 ^e^
10 mg L^−1^	0.035 ± 0.002 ^e^	3.066 ± 0.007 ^e^	0.114 ± 0.005 ^e^	5.735 ± 0.033 ^f^	0.843 ± 0.023 ^f^	0.102 ± 0.011 ^a,b^	3.215 ± 0.013 ^e^	6.578 ± 0.056 ^f^
50 mg L^−1^	nd	2.345 ± 0.006 ^f^	0.027 ± 0.002 ^f^	3.921 ± 0.013 ^g^	0.482 ± 0.002 ^b^	0.032 ± 0.002 ^f^	2.373 ± 0.008 ^f^	4.403 ± 0.011 ^g^

3-CQA—neochlorogenic acid; 5-CQA—chlorogenic acid; 4-CQA—cryptochlorogenic acid; 3,5- and 4,5-DCQA—3,5- and 4,5-dicaffeoylquinic acids; CQAs and DCQAs—total amount of mono- and dicaffeoyl quinic acids; values are mean ± SD (from three measurements). Values with different letters in the columns are significantly different (*p* ≤ 0.05), according to Tukey’s test; nd—not detected.

## Data Availability

The original contributions presented in this study are included in the article. Further inquiries can be directed to the corresponding author.

## Supplementary Materials

The following supporting information can be downloaded at: https://www.mdpi.com/article/10.3390/metabo15090579/s1. Figure S1: UV spectra of solutions of IAA, M6, and M6+IAA; Table S1: Linear ranges, regression equations, correlation coefficients (R^2^), limits of detection (LOD), and limits of quantification (LOQ) of stevioside and rebaudioside A; Figure S2: Calibration curves of stevioside and rebaudioside A; Figure S3: HPLC chromatograms of stevioside, rebaudioside A, and selected samples at 210 nm; Table S2: Linear ranges, regression equations, correlation coefficients (R^2^), limits of detection (LOD), and limits of quantification (LOQ) of chlorogenic acid, 3,5-dicaffeoylquinic acid, 4,5-dicaffeoylquinic acid, and quercitrin; Figure S4: Calibration curves of chlorogenic acid, 3,5-dicaffeoylquinic acid, 4,5-dicaffeoylquinic acid, and quercitrin; Figure S5: HPLC chromatograms of the standard mixture of CQAs and selected samples at 325 nm; Figure S6: HPLC chromatograms of quercitrin and selected samples at 350 nm; Figure S7: *Stevia rebaudiana* plantlets, grown on MS nutrient medium supplemented with 100 mg L^−1^ M6.

## References

[1] da Silva T.F.O., Ferrarezi A.A., da Silva Santos É., Ribeiro S.T.C., de Oliveira A.J.B., Gonçalves R.A.C. (2025). Bioactivities and biotechnological tools for obtaining bioactive metabolites from *Stevia rebaudiana*. Food Sci. Biotechnol..

[2] Khalil S.A., Zamir R., Ahmad N. (2014). Selection of suitable propagation method for consistent plantlets production in *Stevia rebaudiana* (Bertoni). Saudi J. Biol. Sci..

[3] Ghose A.K., Abdullah S.N.A., Md Hatta M.A., Megat Wahab P.E. (2022). In vitro regeneration of stevia (*Stevia rebaudiana* Bertoni) and evaluation of the impacts of growth media nutrients on the biosynthesis of steviol glycosides (SGs). Agronomy.

[4] Yücesan B., Mohammed A., Büyükgöçmen R., Altuğ C., Kavas Ö., Gürel S., Gürel E. (2016). In vitro and ex vitro propagation of *Stevia rebaudiana* Bertoni with high Rebaudioside-A content—A commercial scale application. Sci. Hortic..

[5] Abdullateef R.A., Osman M. (2012). Effects of stem cutting types, position and hormonal factors on rooting in *Stevia rebaudiana* Bertoni. J. Agric. Sci..

[6] Rodriguéz-Páez L.A., Pineda-Rodriguez Y.Y., Pompelli M.F., Jimenez-Ramirez A.M., Genes-Avilez O.J., Jaraba-Navas J.d.D., Jarma-Orozco A., Combatt-Caballero E., Oviedo Zumaqué L.E., Suarez-Padron I.E. (2024). Micropropagation Protocols for Three Elite Genotypes of *Stevia rebaudiana* Bertoni. Horticulturae.

[7] Taha R.A., Hendawy S., Hussien M.S., Mehareb E. (2025). Steviol Glycosides Induced in Vitro from Three Stevia Genotypes Successfully Planted in Egypt. Egypt J. Chem..

[8] Hendawey M.H., Abo El Fadl R.E. (2014). Biochemical studies on the production of active constituents in *Stevia rebaudiana* L. callus. Glob. J. Biochem. Biotechnol..

[9] Alizadeh S., Dumanoğlu H. (2022). The effects of zinc oxide nanoparticles loaded with IAA and IBA on *in vitro* rooting of apple microcuttings. Turk. J. Agric. For..

[10] Gerdakaneh M., Mozafari A.-A., Sioseh-Mardah A., Sarabi B. (2011). Effects of different amino acids on somatic embryogenesis of strawberry (*Fragaria × ananassa* Duch.). Acta Physiol. Plant..

[11] Blomstrand E., Eliasson J., Karlsson H.K., Kohnke R. (2006). Branched-chain amino acids activate key enzymes in protein synthesis after physical exercise. J. Nutr..

[12] Upmeier B., Thomzik J.E., Barz W. (1988). Nicotinic acid-N-glucoside in heterotrophic parsley cell suspension cultures. Phytochemistry.

[13] Zheng X.Q., Nagai C., Ashihara H. (2004). Pyridine nucleotide cycle and trigonelline (N-methylnicotinic acid) synthesis in developing leaves and fruits of *Coffea arabica*. Physiol. Plant..

[14] Noctor G., Queval G., Gakière B. (2006). NAD(P) synthesis and pyridine nucleotide cycling in plants and their potential importance in stress conditions. J. Exp. Bot..

[15] Waller G.R., Yang K.S., Gholson R.K., Hadwiger L.A., Chaykin S. (1966). The pyridine nucleotide cycle and its role in the biosynthesis of ricinine by *Ricinus communis* L.. J. Biol. Chem..

[16] Frost G.M., Yang K.S., Waller G.R. (1967). Nicotinamide adenine dinucleotide as a precursor of nicotine in *Nicotiana rustica* L.. J. Biol. Chem..

[17] Gholson H.R. (1966). The pyridine nucleotide cycle. Nature.

[18] Wagner R., Feth F., Wagner K.G. (1986). Regulation in tobacco callus of enzyme activities of the nicotine pathway. II. The pyridine-nucleotide cycle. Planta.

[19] Wagner R., Feth F., Wagner K.G. (1986). The pyridine-nucleotide cycle in tobacco: Enzyme activities for the recycling of NAD. Planta.

[20] Datta C., Basu P.S. (2000). Indole acetic acid production by a Rhizobium species from root nodules of a leguminous shrub, Cajanus cajan. Microbiol. Res..

[21] Röck-Okuyucu B., Bayraktar M., Akgun I.H., Gurel A. (2016). Plant growth regulator effects on *in vitro* propagation and stevioside production in *Stevia rebaudiana* Bertoni. Hortic. Sci..

[22] Sivaram L., Mukundan U. (2003). *In vitro* culture studies on *Stevia rebaudiana*. Vitr. Cell. Dev. Biol.-Plant.

[23] Zayova E., Stancheva I., Geneva M., Petrova M., Dimitrova L. (2013). Antioxidant activity of *in vitro* propagated *Stevia rebaudiana* Bertoni plants of different origins. Turk. J. Biol..

[24] Soliman H.I.A., Metwali E.M.R., Almaghrabi O.A.-H. (2014). Micropropagation of *Stevia rebaudiana* Betroni and assessment of genetic stability of *in vitro* regenerated plants using inter simple sequence repeat (ISSR) marker. Plant Biotechnol..

[25] Rokosa M.T., Kulpa D. (2019). Micropropagation of *Stevia rebaudiana* plants. Cienc. Rural..

[26] Tiwari K., Tripathi S., Mahra S., Mathew S., Rana S., Tripathi D.K., Sharma S. (2024). Carrier-based delivery system of phytohormones in plants: Stepping outside of the ordinary. Physiol. Plant..

[27] Krishnraj C., Ramachandran R., Mohan K., Kalaichelvan P. (2012). Optimization for rapid synthesis of silver nanoparticle and its effect on phytopathogenic fungi. Spectrochim. Acta.

[28] Karakecili A., Korpayev S., Dumanoglu H., Alizadeh S. (2019). Synthesis of indole-3-acetic acid and indole-3-butyric acid loaded zinc oxide nanoparticles: Effects on rhizogenesis. J. Biotechnol..

[29] Thangavelu R.M., Gunasekaran D., Jesse M.I., Su M.R., Sundarajan D., Krishnan K. (2018). Nanobiotechnology approach using plant rooting hormone synthesized silver nanoparticle as “nanobulets” for the dynamic applications in horticulture—An *in vitro* and ex vitro study. Arab. J. Chem..

[30] Sichanova M., Geneva M., Petrova M., Miladinova-Georgieva K., Kirova E., Nedev T., Tsekova D., Iwanov I., Dochev K., Ivanova V. (2022). Improvement of *Stevia rebaudiana* Bertoni *in vitro* propagation and steviol glycoside content using aminoacid silver nanofibers. Plants.

[31] Tsekova D.S., Stoyanova V.B. (2009). Microstructure of new metal-organic gels obtained by low-molecular gelators. Bulg. Chem. Commun..

[32] Ashwell G., Neufeld E.F., Ginsburg V. (1966). New colorimetric methods of sugar analysis. Methods in Enzymology.

[33] Bergs D., Burghoff B., Joehnck M., Martin G., Schembecker G. (2012). Fast and isocratic HPLC-method for steviol glycosides analysis from *Stevia rebaudiana* leaves. J. Verbrauch. Lebensm..

[34] Sichanova M., Geneva M., Petrova M., Miladinova-Georgieva K., Kirova E., Nedev T., Tsekova D., Ivanova V., Trendafilova A. (2023). Influence of the abiotic elicitors Ag salts of aspartic acid derivatives, self-organized in nanofibers with monomeric and dimeric molecular structures, on the antioxidant activity and stevioside content in micropropagated *Stevia rebaudiana* Bert. Plants.

[35] Hristozkova M., Geneva M., Stancheva I., Iliev I., Azcón-Aguilar C. (2017). Symbiotic association between golden berry (*Physalis peruviana*) and arbuscular mycorrhizal fungi in heavy metal-contaminated soil. J. Plant Prot. Res..

[36] Giannopolitis C.N., Reis S.K. (1997). Superoxide dismutase: I. Occurrence in higher plants. Plant Physiol..

[37] Upadhyaya A., Sankhla D., Davis T., Sankhla N., Smith B. (1985). Effect of paclobutrazol on the activities of some enzymes of activated oxygen metabolism and lipid peroxidation in senescing soybean leaves. J. Plant Physiol..

[38] Nakano Y., Asada K. (1987). Purification of ascorbate peroxidase in spinach chloroplasts: Its inactivation in ascorbate-depleted medium and reactivation by monodehydroascorbate radical. Plant Cell Physiol..

[39] Lagrimini M., Gingas V., Finger F., Rothstein S., Liu T.-T.Y. (1997). Characterization of antisense transformed plants deficient in the tobacco anionic peroxidase. Plant Physiol..

[40] Bradford M.M. (1976). A rapid and sensitive method for the estimation of microgram quantities of protein utilizing the principle of protein-dye binding. Anal. Biochem..

[41] Alexieva V., Sergiev I., Mapelli S., Karanov E. (2001). The effect of drought and ultraviolet radiation on growth and stress markers in pea and wheat. Plant Cell Environ..

[42] Kramer G.F., Norman H., Krizek D., Mirecki R. (1991). Influence of UV-B radiation on polyamines, lipid peroxidation and membrane lipids in cucumber. Phytochemistry.

[43] Bates L., Waldren R., Teare I.D. (1973). Rapid determination of free proline for water-stress studies. Plant Soil.

[44] Ellman G.l. (1959). Tissue sulphydryl groups. Arch. Biochem. Biophys..

[45] Ludwig-Müller J., Niemi K., Scagel C. (2009). Molecular basis for the role of auxins in adventitious rooting. Adventitious Root Formation of Forest Trees and Horticultural Plants—From Genes to Applications.

[46] Muslihatin W., Febriawan Z., Nasution A.M.T., Patrialoka S.N., Pratama I.P.E.W., Aisyah P.Y., Jadid N., Fatmawati S., Antika T.R., Shovitri M. (2023). Morphological and physiological characteristic of *Stevia rebaudiana* Bertoni stem cuttings under 3-indoleacetic acid (IAA) treatment. Agriculture (Poľnohospodárstvo).

[47] Li S.W., Xue L., Feng H. (2009). IBA induced changes in antioxidant enzymes during adventitious rooting in mung bean seedlings. Environ. Exp. Bot..

[48] Javed R., Yucesan B., Zia M., Gurel E. (2017). Differential effects of plant growth regulators on physiology, steviol glycosides content, and antioxidant capacity in micropropagated tissues of *Stevia rebaudiana*. Biologia.

[49] Libik-Konieczny M., Capecka E., Tuleja M., Konieczny R. (2021). Synthesis and production of steviol glycosides: Recent research trends and perspectives. Appl. Microbiol. Biotechnol..

[50] Wang H., Shan X., Wen B., Owens G., Fang J., Zhang S. (2007). Effect of indole-3-acetic acid on lead accumulation in maize (*Zea mays* L.) seedlings and the relevant antioxidant response. Environ. Exp. Bot..

[51] Gong Q., Li Z., Wang L., Dai T., Kang Q., Niu D. (2019). Exogenous of indole-3-acetic acid application alleviates copper toxicity in spinach seedlings by enhancing antioxidant systems and nitrogen metabolism. Toxics.

[52] Ma C., Yuan S., Xie B., Li Q., Wang Q., Shao M. (2022). IAA plays an important role in alkaline stress tolerance by modulating root development and ROS detoxifying systems in rice plants. Int. J. Mol. Sci..

[53] Radić S., Vujčić V., Glogoški M., Radić-Stojković M. (2016). Influence of pH and plant growth regulators on secondary metabolite production and antioxidant activity of *Stevia rebaudiana* (Bert). Period. Biol..

[54] Barroso M., Barros L., Rodrigues M.A., Sousa M.J., Santos-Buelga C., Ferreira I.C.F.R. (2016). *Stevia rebaudiana* Bertoni cultivated in Portugal: A prospective study of its antioxidant potential in different conservation conditions. Ind. Crops Prod..

[55] Barroso M.R. (2019). *Stevia rebaudiana* Bertoni Cultivated in the Field and Obtained by In Vitro Micropropagation: A Prospective Study of the Antioxidant Potential in Different Culture and Storage Conditions. Ph.D. Thesis.

[56] Moat A.G., Foster J.W., Dolphin D., Avramovic O., Poulson R. (1987). Biosynthesis and salvage pathways of pyridine nucleotides. Pyridine Nucleotide Coenzymes. Chemical, Biochemical, and Medical Aspects, Part B.

[57] Ashihara H., Stasolla C., Yin Y., Loukanina N., Thorpe T.A. (2005). De novo and salvage biosynthetic pathways of pyridine nucleotides and nicotinic acid conjugates in cultured plant cells. Plant Sci..

[58] Ahmad Z., Bashir K., Matsui A., Tanaka M., Sasaki R., Oikawa A., Hirai M.Y., Chaomurilege, Zu Y., Kawai-Yamada M. (2021). Overexpression of nicotinamidase 3 (NIC3) gene and the exogenous application of nicotinic acid (NA) enhance drought tolerance and increase biomass in Arabidopsis. Plant Mol. Biol..

[59] Khurshid N., Bukhari M.A., Ahmad T., Ahmad Z., Jatoi W.N., Abbas S.M., Latif A., Raza A., Aurangzaib M., Hashem A. (2023). Exogenously applied nicotinic acid alleviates drought stress by enhancing morpho-physiological traits and antioxidant defense mechanisms in wheat. Ecotoxicol. Environ. Saf..

[60] Dalvand S., Mumivand H., Zahedi B., Nia A.E. (2024). The effect of foliar application of glutamic acid, aspartic acid, and nicotinic acid on the growth, yield, and morpho-physiological and biochemical characteristics of fenugreek (*Trigonella foenum-graecum* L.). Plant Prod..

[61] Yu Z., Xu Y., Zhu L., Zhang L., Liu L., Zhang D., Li D., Wu C., Huang J., Yang G. (2020). The Brassicaceae-specific secreted peptides, STMPs, function in plant growth and pathogen defense. J. Integ. Plant Biol..

[62] Ahkami A.H., Melzer M., Ghaffari M.R., Pollmann S., Ghorbani J.M., Shahinnia F., Hajirezaei M.R., Druege U. (2013). Distribution of indole-3-acetic acid in Petunia hybrida shoot tip cuttings and relationship between auxin transport, carbohydrate metabolism and adventitious root formation. Planta.

[63] Wu K., Liang X., Zhang X., Yang G., Wang H., Xia Y., Ishfaq S., Ji H., Qi Y., Guo W. (2024). Metabolomics analysis reveals enhanced salt tolerance in maize through exogenous valine-threonine-isoleucine-aspartic acid application. Front. Plant Sci..

[64] Singh B.K., Singh B.K. (1998). Biosynthesis of valine, leucine, and isoleucine. Plant Amino Acids.

[65] Scheel D., Casida J.E. (1985). Sulfonylurea herbicides: Growth inhibition in soybean cell suspension cultures and in bacteria correlated with block in biosynthesis of valine, leucine, or isoleucine. Pestic. Biochem. Physiol..

[66] Ahmad A., Hashmi S.S., Palma J.M., Corpas F.J. (2022). Influence of metallic, metallic oxide, and organic nanoparticles on plant physiology. Chemosphere.

