# Supplementary material for: Application of Organic Nanofibers to Boost Specialized Metabolite Production and Antioxidant Potential in Stevia rebaudiana In Vitro Cultures
Source: Metabolites. 2025 Aug 29;15(9):579. doi: 10.3390/metabo15090579 (PMC12471799; doi:10.3390/metabo15090579)
Supplement: Supplementary file 1 [file metabolites-15-00579-s001.zip › metabolites-3834997-supplementary.pdf]

Application of Organic Nanofibers to Boost Specialized Metabolite Production and Antioxidant Potential in *Stevia rebaudiana in vitro* Cultures

Maria Geneva [1], Antoaneta Trendafilova [2], Kamelia Miladinova-Georgieva [1,*], Mariana Sichanova [1], Daniela Tsekova [3], Viktoria Ivanova [2], Elisaveta Kirova [1], and Maria Petrova [1]

SUPPLEMENTARY MATERIAL

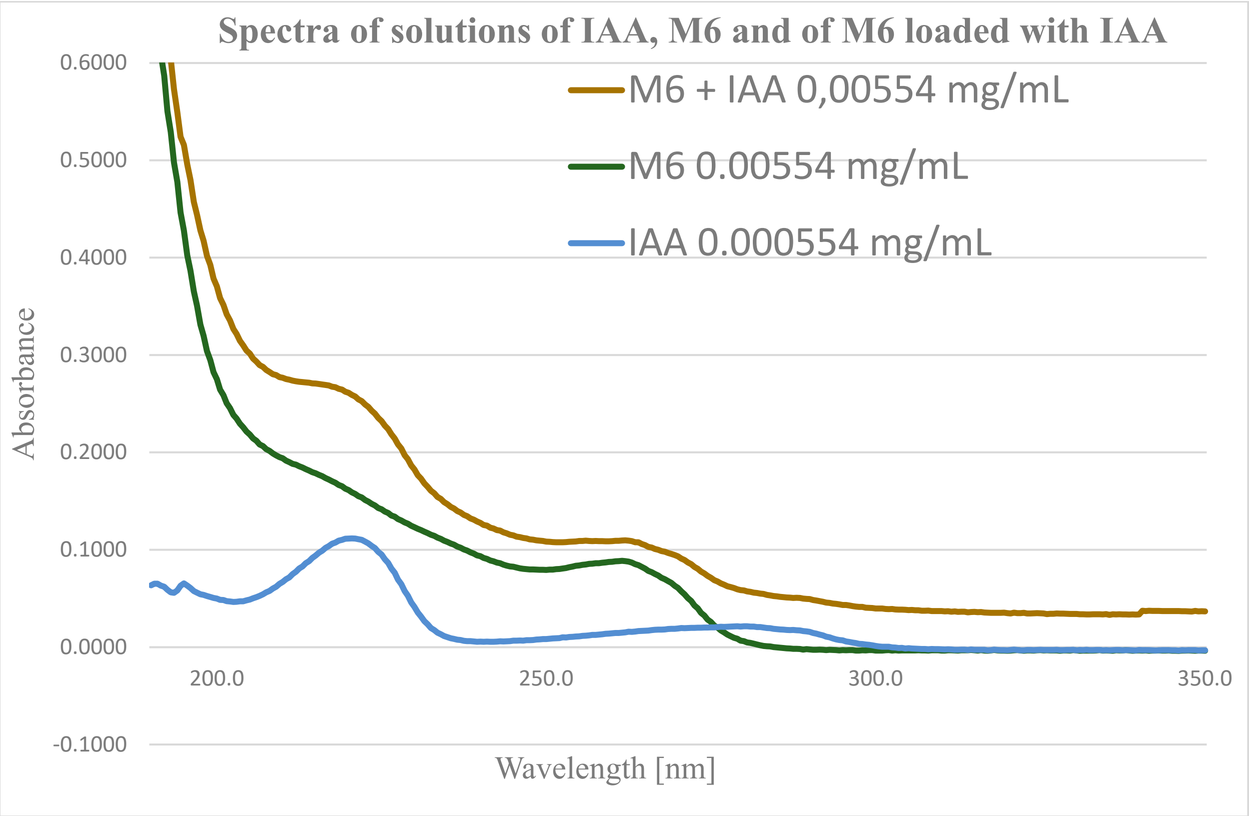

Figure S1. UV spectra of solutions of IAA, M6 and of M6+IAA

Table S1. Linear ranges, regression equations, correlation coefficients (R^2), limits of detection (LOD) and limits of quantification (LOQ) of stevioside and rebaudioside A

Compound	Linear range (mg/mL)	Regression equation	R^2	LOD (mg/mL)	LOQ (mg/mL)
Stevioside	0.0625–1.00	y = 1163756x + 120.33	0.9998	0.019	0.058
Rebaudioside A	0.0625–1.00	y = 889910x + 995.08	0.9999	0.017	0.052

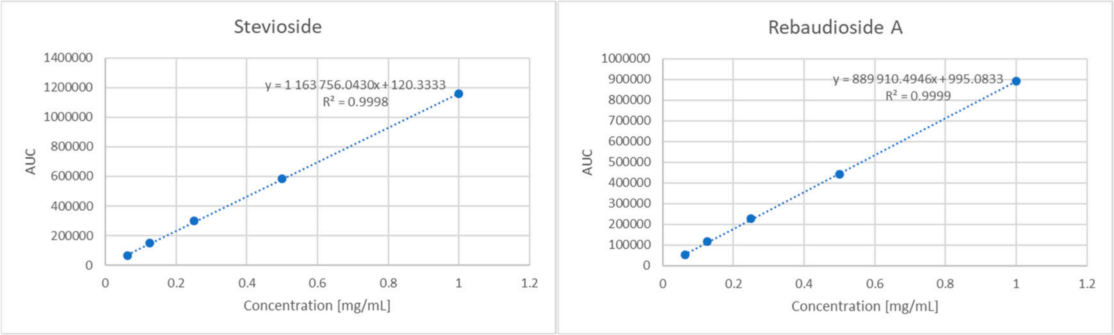

Figure S2. Calibration curves of stevioside and rebaudioside A

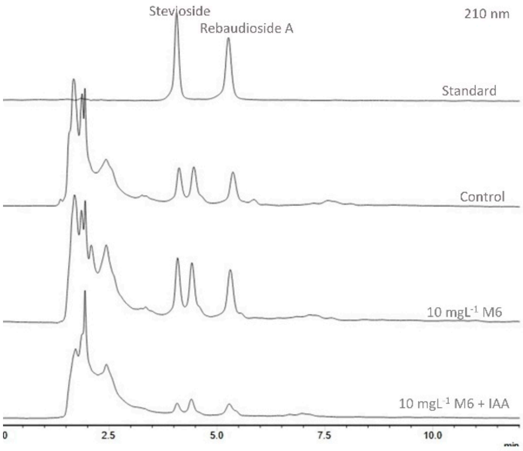

Figure S3. HPLC chromatograms of stevioside, rebaudioside A, and selected samples at 210 nm

Table S2. Linear ranges, regression equations, correlation coefficients (R^2), limits of detection (LOD) and limits of quantification (LOQ) of chlorogenic acid, 3,5-dicaffeoylquinic acid, 4,5-dicaffeoylquinic acid and quercitrin

Compound	Linear range (mg/mL)	Regression equation	R^2	LOD (mg/mL)	LOQ (mg/mL)
Chlorogenic acid	0.027–0.423	y = 5341935.17+ 4640.8	0.9996	0.012	0.035
3,5-Dicaffeoylquinic acid	0.026–0.410	y = 6825336.20x - 10302.95	0.9995	0.013	0.040
4,5-Dicaffeoylquinic acid	0.022–0.363	y = 6964715.33x + 2845.73	0.9997	0.009	0.028
Quercitrin	0.004–0.057	y = 3438384.16x - 1031.79	0.0994	0.002	0.006

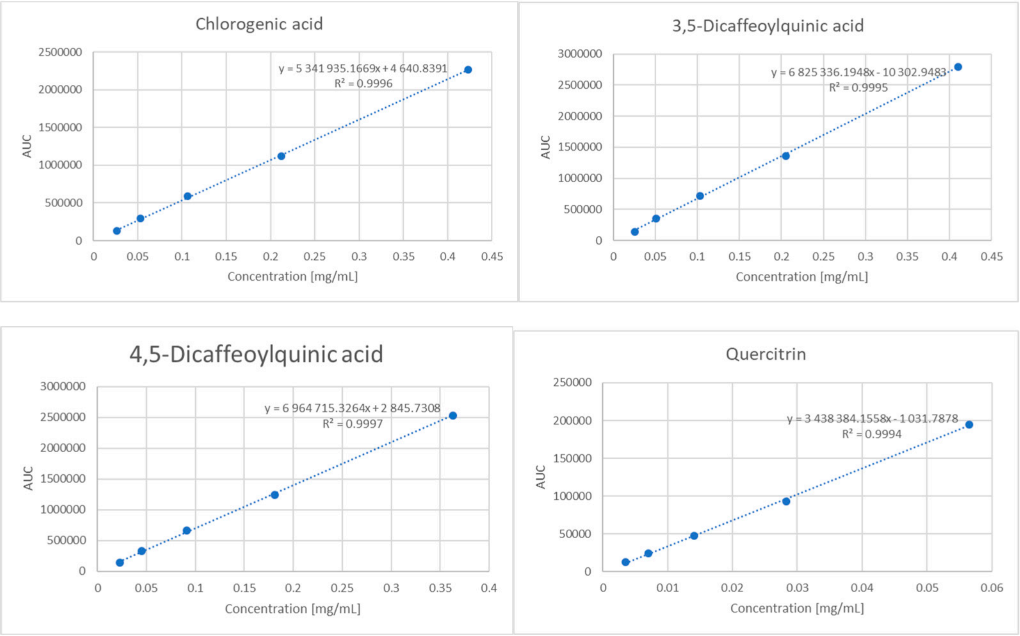

Figure S4. Calibration curves of chlorogenic acid, 3,5-dicaffeoylquinic acid, 4,5-dicaffeoylquinic acid and quercitrin

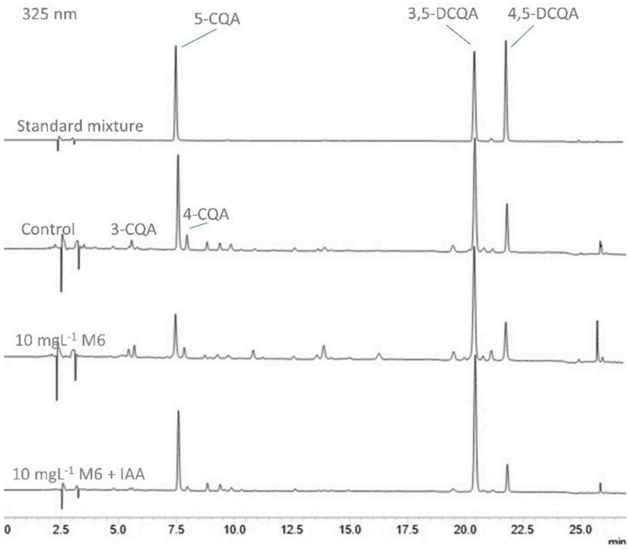

Figure S5. HPLC chromatograms of the standard mixture of CQAs and selected samples at 325 nm

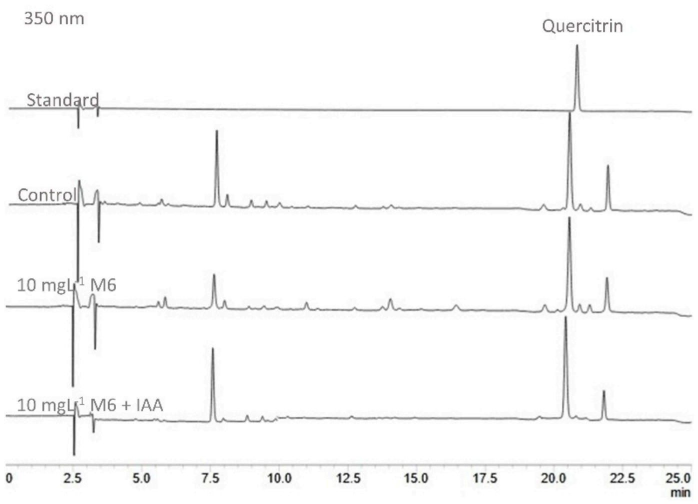

Figure S6. HPLC chromatograms of quercitrin and selected samples at 350 nm

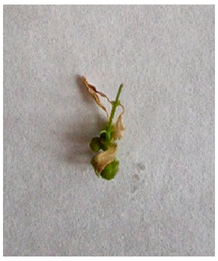

Figure S7. *Stevia rebaudiana* plantlets, grown on MS nutrient medium supplemented with 100 mg L^{-1} M6
